# Zn(II) binding to pramlintide results in a structural kink, fibril formation and antifungal activity

**DOI:** 10.1038/s41598-022-24968-y

**Published:** 2022-11-29

**Authors:** Dorota Dudek, Emilia Dzień, Joanna Wątły, Agnieszka Matera-Witkiewicz, Aleksandra Mikołajczyk, Agata Hajda, Joanna Olesiak-Bańska, Magdalena Rowińska-Żyrek

**Affiliations:** 1 Faculty of Chemistry, University of Wrocław, F. Joliot-Curie 14, 50-383 Wrocław, Poland; 2 Screening of Biological Activity Assays and Collection of Biological Material Laboratory, Faculty of Pharmacy, Wrocław Medical University Biobank, Wrocław Medical University, Wrocław, Poland; 3 Faculty of Chemistry, Wrocław University of Science and Technology, Wyb. Wyspiańskiego 27, 50-370 Wrocław, Poland

**Keywords:** Chemical biology, Chemistry, Analytical chemistry, Coordination chemistry, Inorganic chemistry

## Abstract

The antimicrobial properties of amylin, a 37-amino acid peptide hormone, co-secreted with insulin from the pancreas, are far less known than its antidiabetic function. We provide insight into the bioinorganic chemistry of amylin analogues, showing that the coordination of zinc(II) enhances the antifungal properties of pramlintide, a non-fibrillating therapeutic analogue of amylin. Zinc binds to the N-terminal amino group and His18 imidazole, inducing a kink in the peptide structure, which, in turn, triggers a fibrillization process of the complex, resulting in an amyloid structure most likely responsible for the disruption of the fungal cell.

## Introduction

Amylin (islet amyloid polypeptide, IAPP) is a 37-amino acid peptide hormone, co-secreted with insulin from β-cells of the pancreatic Langerhans islets^1^.The native form of amylin is amidated at the C-terminus and has a disulfide bridge between Cys2 and Cys7. Together with insulin, it is involved in blood-glucose level control by inhibiting the production of glucagon and the release of glucose from the liver^2^. Amylin is the main component of protein aggregates that accumulate in Langerhans islets of type 2 diabetes (T2D) patients, and due to its tendency of misfolding and fibril formation, it plays crucial role in β-cell membrane disruption^3–5^.

**Figure 1:**
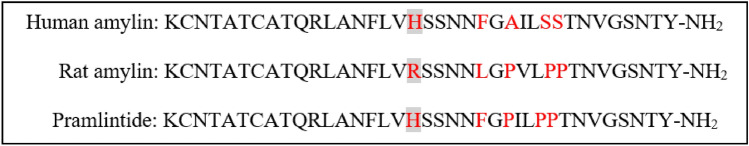
A comparison of the native sequence of human amylin, rat amylin and pramlintide. Sequential differences are marked in red.

Human amylin is one of the most amyloidogenic peptides, while both its rat analogue, and its soluble, clinically used analogue, pramlintide, which differ only by 5 and 3 residues, respectively, show no such tendency (Fig. 1). According to MD simulations, the presence of Pro in position 28 prevents H-bonding of Ile26, Leu27 and aromatic amino acid residues and thus inhibits peptide fibrilization^6,7^. The H18R substitution in rat amylin is believed to be crucial for the metal binding abilities of both analogues. There is a number of studies trying to elucidate the role of metal ions, in particular Zn(II) and Cu(II), in the fibrillization of amylin, as well as in T2D progression^8–11^.

Zinc(II) is stored together with amylin inside pancreatic β-cells, in one of the highest concentrations found in the human body (10–20 mM)^12^. Both Zn(II) and Cu(II) play a role in glycemic regulation and zinc(II) deficiency is common among T2D patients, making these metals a ‘hot topic’ in the bioinorganic chemistry of amylin. According to in vitro studies, zinc(II) may have a dual effect on amylin aggregation, either inhibiting^13^ or accelerating^14–16^ the process, depending on its concentration. NMR studies performed by Brender et al. indicate His18 acts as an anchoring site for zinc(II) in the monomeric form of human amylin^15^. The non-fibrillating pramlintide and its membrane disrupting fragment, amylin_1-19_, coordinate zinc(II) by the imidazole nitrogen and the N-terminal amine group of Lys1, imposing a kink in the peptide backbone^11,17^.

The protective role of Cu(II) in amylin-induced cytotoxicity is shown in a number of studies, some of which suggest that its presence prolongs the lag phase of fibril formation by increasing the activation energy of this process or by inhibiting dimer formation, and therefore limiting β structure formation^9,18–20^. Also our previous studies support the inhibitory role of Cu(II) in pramlintide aggregation and show that the N-terminal regions of both pramlintide and rat amylin are involved in Cu(II) binding; the imidazole of His18 is an equally attractive binding site in the case of pramlintide^21^. This is in good agreement with the results obtained by Sanchez–Lopez’s group, who show that in several amylin fragments Cu(II) binds to the imidazole nitrogen of His18^22^. The undeniable role of His18 in copper binding is also seen in our previous studies on the amylin_1-19_ fragment^17^ and in the work of Magri et al. on the R18H substituted version of rat amylin^23^.

The much less-known property of amylin (besides its function of glycemic control) is its recently described antimicrobial activity, which formally allows to classify amylin to the family of antimicrobial peptides (AMPs)^24^. In fact, there are several molecular features that link amyloid peptides to AMPs. First, while amyloids are mainly associated with neurodegenerative diseases and AMPs are host-defense peptides involved in innate immunity, in both groups, cytotoxicity is mainly caused by membrane disruption. It is the case in uperin 3.5, an amphibian AMP, which adopts an α-helical structure in membrane-like environments, and forms amyloid fibrils rapidly in solution at neutral pH^25^ or in the case of *β*-hairpin protegrin-1 (PG-1), a broad-spectrum AMP isolated from porcine leukocytes^26^; which forms membrane channels, that exhibit common structural and biological properties to amyloid *β –* peptide^27,28^. A number of amyloid peptides, such as human prion protein^29^, amyloid β^30^ and human amylin, also show antimicrobial properties. An interesting fact was discovered by Last et al., who found that amylin is able to cause liposomal leakage, yet it is far more effective when applied as a mixture with another AMP – magainin 2. Amylin’s antibacterial properties against *Paracoccus denitrificans* also increased dramatically when combined with magainin 2^31^.

Here, we present a link between the coordination chemistry, morphology and antimicrobial activity of Zn(II) and Cu(II) complexes of amylin analogues, rat amylin, pramlintide and N-terminally acetylated pramlintide.

## Results and discussion

Zn(II)-rat amylin complexes are equimolar, according to mass spectrometry (Fig. S1)—four most intensive signals can be assigned to the free ligand sodium adduct (*m/z* = 658.0; *z* = 6 +), the free ligand potassium adduct (*m*/*z* = 660.7, *z* = 6 +), the rat amylin zinc complex (*m/z* = 664.5; *z* = 6 +) and the free ligand adduct with two potassium atoms (*m/z* = 667.0; *z* = 6 + ; Fig. S2A).

It is worth to notice that the signals from the zinc-rat amylin complex are overlapped with the two sodium adduct of rat amylin (Fig. S2B).

### Potentiometric measurements

For rat amylin, 3 protonation constants derived from the free N-terminal amine group and the tyrosine and lysine side groups were determined by potentiometric titrations (Table S1).

In its Zn(II) complex, the first observed form starts to appear at pH 6.5 (Fig. S3). The maximum of the ZnL form is at pH about 8, where the N-terminal amine nitrogen atom is most likely already coordinated, and, in addition, two coordinated water molecules have been deprotonated. The loss of one more proton leads to the formation of the ZnH_-1_L form, which is most likely formed by the deprotonation of a third water molecule. Another complex form, ZnH_-3_L is formed by deprotonation of tyrosine and lysine side groups, which does not affect the coordination mode of the metal.

**Figure 2:**
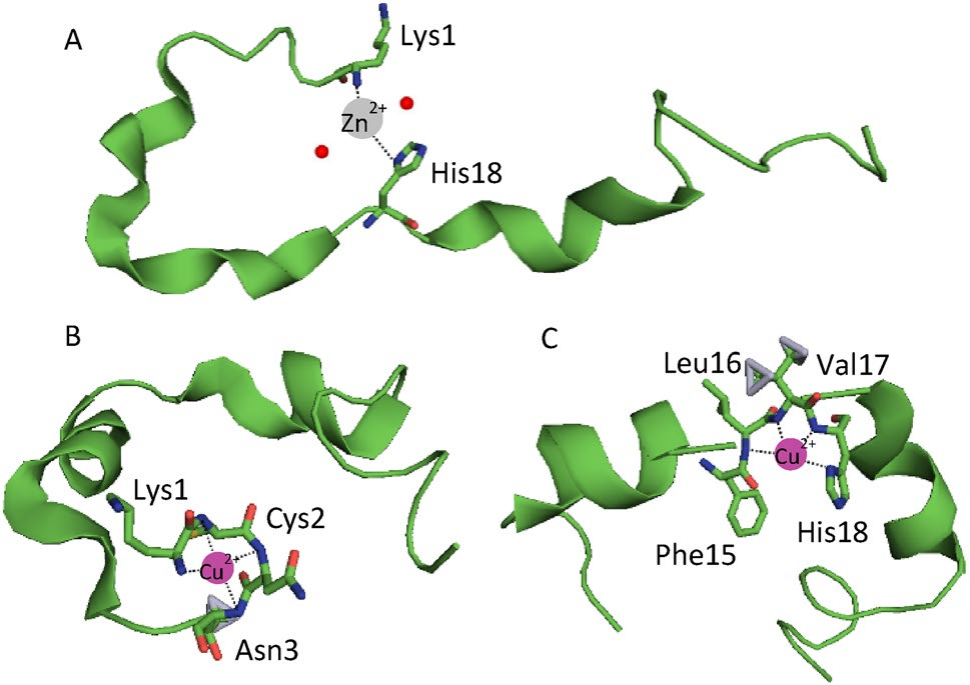
Established coordination modes for the (**A**) Zn(II)-pramlintide complex above pH 7.4, and Cu(II)-pramlintide complex. At pH 7.4 Cu(II) may be coordinated to either (**B**) the N-terminal amine and three neighbouring amide nitrogens or (**C**) the His18 imidazole nitrogen and three neighbouring amide nitrogens. The structures are based on a Phyre2 simulation^33^ and the figure was generated by PyMOL^34^.

Stability constants for the Zn(II)-pramlintide complex are published in our previous work^11^. At pH 7.4, Zn(II) binding to rat amylin occurs via the N-terminal amine nitrogen, while in the Zn(II)-pramlintide complex, zinc binds to pramlintide’s N-terminal amine and to the His18 imidazole, resulting in an {N_im_, NH_2_} type of coordination (Figs. 2A and S1). The fundamental difference in binding is a result of the H18R substitution.

Rat amylin binds Cu(II) via the N-terminal amine and three neighbouring amide nitrogens (toward the C-terminus)^21^, while the N-terminally acetylated pramlintide binds Cu(II) to the His18 imidazole nitrogen and three preceding amide nitrogens^32^. Cu(II) binding to pramlintide is a combination of the two coordination modes described above, which are in equilibrium with each other: Cu(II) binds either to the N-terminal amine and the next three amides (Fig. 2B), or the His18 imidazole and three preceding amide nitrogens (Fig. 2C)^21^.

Do these binding modes have an impact on the antimicrobial activity of the amylin-related complexes? To answer the question, we carried out antimicrobial susceptibility testing of rat amylin, pramlintide and Ac-pramlintide on two bacterial (*Escherichia coli* ATCC 25,922, MRSA *Staphylococcus aureus* ATCC 43,300) and one fungal (*Candida albicans* ATCC 10,231) strain. The minimal inhibitory concentration (MIC) was checked for all compounds and their complexes with Cu(II) and Zn(II) (according to the ISO 20,776–1:2019^35^ and ISO 16,256:2012^36^). No activity against bacterial strains (and also no toxicity against regular cell lines) was detected in the tested concentration range. Based on the results obtained by Lan Wang et al.^24^ it can be concluded that native amylin is more potent against *E. coli* than *S. aureus*, however, we can’t confirm a similar behavior of our amylin analogues, because the highest concentration in our tested range was lower than the one used by the authors (5 μM)^24^.

Most interestingly, only one of the tested samples – the Zn(II)-pramlintide complex, showed antifungal activity against *C. albicans* (Table 1). The spectrophotometric measurement showed the decrease of the absorbance to 50% as referred to the positive control, what determined the MIC value. Moreover, results of the 2,3,5-triphenyltetrazolium chloride (TTC) experiment show no MBC/MFC behavior, because in the lowest concentration required to kill a particular microbial strain, determined by visual analysis after 24 h incubation with TTC, the TTC did not change colour to pink. To summarize, the Zn-pramlintide complex presents only Minimal Inhibitory Concentration (MIC) against *C. albicans.*

**Table 1:** In vitro antimicrobial activity of rat islet amyloid polypeptide, pramlintide and pramlintide acetate (rat amylin, pramlintide and Ac-pramlintide, respectively) with or without copper/zinc ions, expressed as a minimal inhibitory concentration (MIC) (µg/mL); n/d, not determined. No MBC/MFC activity was observed after performing modified Richard’s methodl^37–39^.

Strain	*Escherichia coli* ATCC 25922	*Staphylococcus aureus ATCC 43300*	*Candida albicans* ATCC 10231
	MIC (µg/mL)	MIC (µg/mL)	MIC (µg/mL)
rat amylin	n/d	n/d	n/d
Cu(II)-rat amylin	n/d	n/d	n/d
Zn(II)-rat amylin	n/d	n/d	n/d
pramlintide	n/d	n/d	n/d
Cu(II)-pramlintide	n/d	n/d	n/d
Zn(II)-pramlintide	n/d	n/d	**256**
Ac-pramlintide	n/d	n/d	n/d
Cu(II)-Ac-pramlintide	n/d	n/d	n/d
Zn(II)-Ac-pramlintide	n/d	n/d	n/d

Significant values are in [bold].

**Figure 3:**
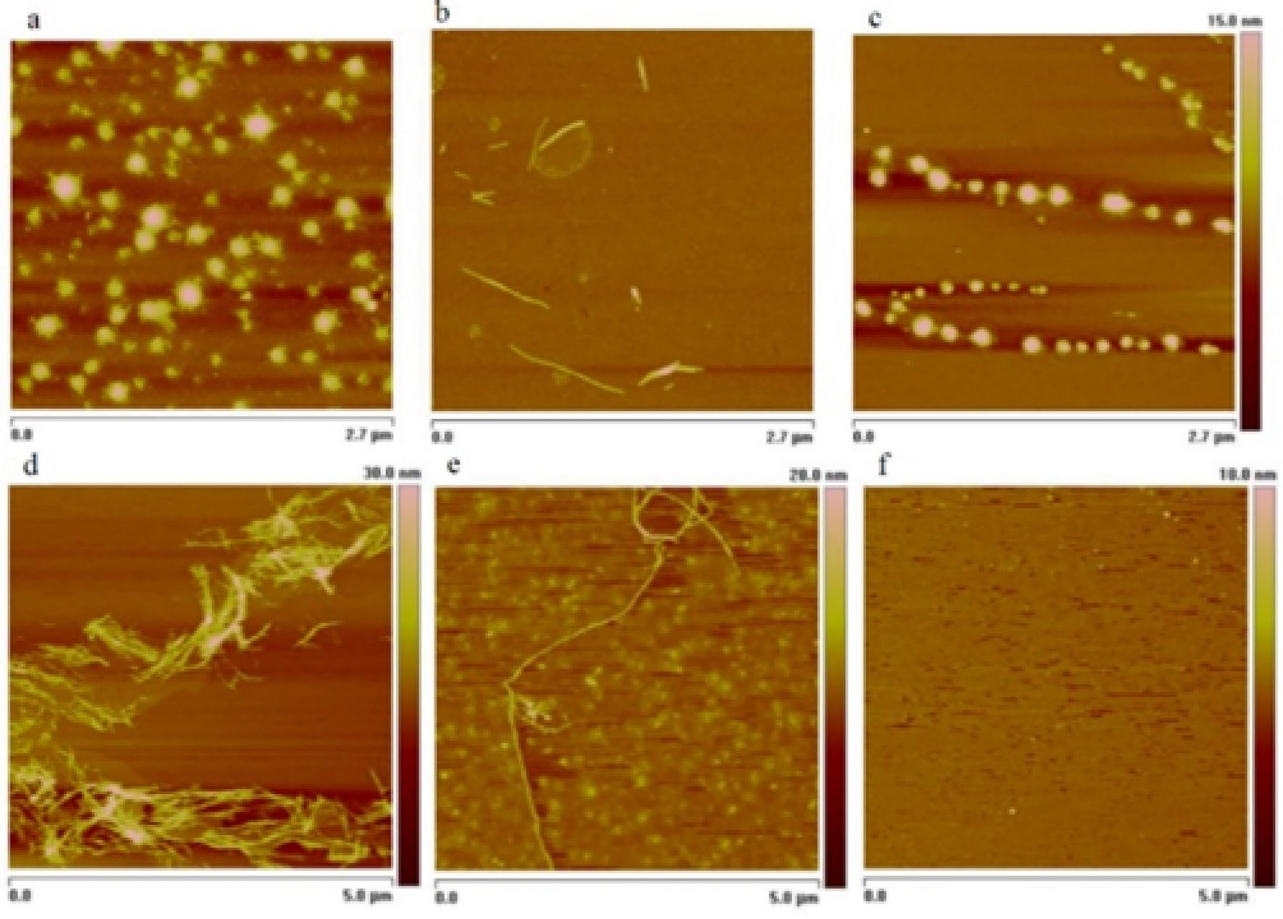
Atomic force microscopy images of pramlintide after 24 h incubation (**a**) without metal ions; (**b**) with Zn(II) ions; (**c**) with Cu(II) ions and after 7 days incubation (**d**) without metal ions; (**e**) with Zn(II) ions; (**f**) with Cu(II) ions.

Why only this one, among all the studied amylin analogues and their Zn(II) and Cu(II) complexes? A literal ‘closer look’ at the morphology of these complexes allowed to answer the question – imaging of the peptides and their complexes was performed under atomic force microscope. Rat amylin, pramlintide and Ac-pramlintide were incubated with and without Zn(II) and Cu(II) ions, and the morphology of the samples was imaged after 24 h and compared with the images of samples before incubation. Amyloid fibrils were observed only for the anticandidal Zn(II)-pramlintide complex, as shown in Fig. 3b. The individual fibrils were 17 nm + / − 3 wide and 4.6 + / − 1.2 high, thus, according to the literature data about the average sizes of fibrils and protofibrils, we have observed both structures in the sample^40,41^. For the pure pramlintide and pramlintide with Cu(II), after 24 h only spherical oligomers were observed with average diameter of 85 + / − 17 nm for pramlintide and 66 + / − 19 for pramlintide with Cu(II), respectively (Fig. 3a,c). Interestingly, longer incubation of samples resulted in amyloid fibril formation not only in the sample with Zn(II), but also in pure pramlintide, whereas no fibrils were observed in the sample with Cu(II) (Fig. 3d–f). The lack of protofibrils or fibrils for pramlintide without metal ions in the first timepoint was observed, while for the system with zinc ions, the fibrils were present already after 24 h, which indicates a catalytic effect of Zn(II). On the other hand, binding of pramlintide to Cu(II) prevented fibrillation, which is in good agreement with our previous studies^21^ and with studies on native amylin, which showed that the inhibitory effect of Cu(II) coordination to the imidazole ring of His18 affects amyloid fibril formation, being related to the sensitivity of histidine to the changing pH of the environment^42^. In the absence of metal ions, the positively charged histidines of native amylin repel each other because of electrostatic interactions, that prevents dimerization. At physiological pH (pH 7.4), amyloid deposits begin to form, which is related to deprotonation of the imidazole ring^43^.

In samples of rat amylin and Ac-pramlintide no fibrils were observed, regardless of addition of metal ions, after both 24 h and 7 days of incubation.

## Conclusion

Among the studied complexes Zn(II)-pramlintide is the only one which is able to simultaneously engage the N-terminal amine group and the His18 imidazole in binding, is amyloidogenic and antifungal. Why? NMR studies reveal that some amyloidogenic peptides (among which amylin is also included) induce membrane damage in two steps – first, oligomers cause leakage, and in the next step, fibrils lead to membrane fragmentation^44,45^. To the best of our knowledge, there is no precisely described process in which amyloids could damage the fungal cell wall–composed mainly of glucans, chitin and glycoproteins, the cell wall is a characteristic structure of fungi that could serve as a primary target for non-specific interactions with fibrils, which could lead to cell wall damage. However, reports of amyloid-based cell wall disruption are becoming more evident in the recent literature–e.g. *C. albicans* treatment with amyloidogenic Aβ peptide variants causes the formation of large agglutinates, showing the antifungal properties of amyloid-β^46^. According to another study, amyloid A protein (SAA1) is able to bind to fungal cell wall adhesin, which promotes cell aggregation in *C. albicans* and induces cell death due to SAA1 hexamer formation, leading to the disruption of the fungal cell wall^47^.

The antifungal properties of Zn(II)-bound pramlintide are a chemically fascinating phenomenon of a metal coordination induced structural change that leads to fibril formation which is possibly relevant to establishing a new mechanism of fungal cell wall disruption. However, one has to keep in mind that this hypothesis, although quite probable, requires further microscopic validation of fibril-induced morphological perturbation of live Candida cells under a variety of conditions.

## Methods

### Materials

The C-protected disulfide-bridged rat amylin, pramlintide and Ac-pramlintide (KCNTATCATQRLANFLVRSSNNLGPVLPPTNVGSNTY-NH_2_, KCNTATATCATQRLANFLVHSSNNFGPILPPTNVGSNTY-NH_2_ and Ac-KCNTATATCATQRLANFLVHSSNNFGPILPPTNVGSNTY-NH_2_) were purchased from KareBayBiochem (USA) (certified purity of 99.30%) and used as received. The purity was checked potentiometrically. The Zn(ClO_4_)_2_·6H_2_O was an extra pure product (Sigma-Aldrich). The carbonate-free stock solution of 0.1 M NaOH was purchased from Merck and then potentiometrically standardized with potassium hydrogen phthalate.

### Mass spectrometry

High-resolution mass spectra was obtained on a BruckerQ-FTMS spectrometer (Bruker Daltonik, Bremen, Germany), equipped with Apollo II electrospray ionization source with an ion funnel. The mass spectrometer was operated both in the positive ion mode. The instrumental parameters were as follows: scan range m/z 300–3000, dry gas–nitrogen, temperature 170 °C, ion energy 5 eV. Capillary voltage was optimized to the highest S/N ratio and it was 4500 V. The small changes of voltage (± 500 V) did not significantly affect the optimized spectra. The samples (Zn(II):ligand in a 0.9:1 stoichiometry, [ligand]tot = 10^−4^ M) were prepared in 1:1 acetonitrile–water mixture at pH 7.4. The variation of the solvent composition down to 5% of acetonitrile did not change the species composition. The sample was infused at a flow rate of 3 μL/min. The instrument was calibrated externally with the Tunemix™ mixture (BrukerDaltonik, Germany) in the quadratic regression mode. Data were processed by using the Bruker Compass DataAnalysis 4.0 program. The mass accuracy for the calibration was better than 5 ppm, enabled together with the true isotopic pattern (using SigmaFit) an unambiguous confirmation of the elemental composition of the obtained complex.

### Potentiometric measurements

Stability constants for proton and Zn(II) complex were calculated from a series of at least three pH-metric titration curves carried out over the pH range 2–11 at T = 298 K in water solution of 4 mM HClO_4_, using a total volume of 3 cm^3^. The potentiometric titrations were performed using a Metrohm Titrando 905 titrator and a Mettler Toledo InLab microglass electrode. The thermostabilized glass-cell was equipped with a magnetic stirring system, a microburet delivery tube and an inlet–outlet tube for argon. Solutions were titrated with 0.1 M carbonate-free NaOH. The electrodes were daily calibrated for hydrogen ion concentration by titrating HClO_4_ with NaOH in the same experimental conditions as above. The purities and the exact concentrations of the ligand solutions were determined by the Gran method^48^. The ligand concentration was 0.5 mM, the Zn(II) to ligand ratio was 0.9:1. HYPERQUAD 2006 program was used for the stability constant calculations^49^. Standard deviations were computed by HYPERQUAD 2006 and refer to random errors only. The speciation and competition diagrams were computed with the HYSS program^50^.

### Antimicrobial activity assay of peptide and peptide-metal ion complex system

Three reference strains from ATCC collection (*Staphylococcus aureus* 43,300, *Escherichia coli* 25,922 and *Candida albicans* 10,231) were used for antimicrobial activity assay. The antimicrobial effect of analysed peptides/complexes was performed according to the standard protocol using microdilution method with spectrophotometric measurement (λ = 580 nm at starting point and after 24 h)^51^ according to the ISO standard 20,776–1:2019^35^, ISO standard 16,256:2012^36^ and modified Richard’s method^37–39^_._

The microdilution method provides information on the decrease in the survival rate of a particular strain as a result of incubation with the tested compound. This is a quantitative method that compares the absorption result of the test samples with the positive control absorption result and calculates the Minimum Inhibitory Concentration.

Stock peptide solutions were prepared in 0.9% TSB four times concentrated. Serial dilutions of ligand/complex solution were made on 96-well microplates in the range between 0.5 and 256 µg/mL. Tryptone Soya Agar (TSA) plates were inoculated with microbial strains from performed stocks. After 24 h/37 °C incubation (for bacteria) or 24 h/25 °C (for fungus), a proper density of bacterial and fungal suspension was prepared using a densitometer (final inoculum 5 × 10^5^ CFU/mL) was prepared in Tryptic Soy Broth (TSB). A positive (TSB + strain) and negative control (TSB) were also included in the test. Spectrophotometric solubility control of each peptide and peptide-metal ion system was also performed. For each strain, the validation process was performed using following antibacterial/antifungal agents: levofloxacin, gentamicin or amphotericin B, according to the EUCAST examination.

MIC was determined from a series of at least three experiments as the lowest concentration of an antimicrobial agent that decreased the measured microbial growth to 50% as referred to positive control. Obtained MIC values were for *Staphylococcus aureus* 43,300: levofloxacin 1 µg/mL, *Escherichia coli* 25,922: gentamicin 2 µg/mL, *Candida albicans* 10,231: amphotericin B 1 µg/mL. Microplates were incubated at 37 ± 1 °C or 25 ± 1 °C for 24 h on the shaker (500 rpm). After this, the spectrophotometric measurement was performed at 580 nm and then 50 μL aliquots of 1% (m/v) 2,3,5-triphenyltetrazolium chloride (TTC) solution were added into each well. 2,3,5-triphenyltetrazolium chloride (TTC) is a commonly used indicator of cellular respiration. In oxidized form, TTC is colourless, while it turns pink after reduction due to reactions in the respiratory chain. MBC/MFC (Minimum Bactericidal/Fungicidal Concentration) can be observed as the lowest concentration required to kill a particular microbial strain, determined by visual analysis after 24 h incubation with TTC. The pink colour indicates the presence of living microorganisms, while the lack of colour indicates that the colonies do not survive. Thanks to both methods, MIC and MBC or MFC can be determined.

### Natural Red cytotoxity assay

For each peptide and peptide-metal ion system, where the antimicrobial activity was determined, a Neutral Red (NR) cytotoxicity assay was performed using human primary renal proximal tubule epithelial cells (RPTEC) from ECACC collection. The experiment was performed according to ISO:10,993 guidelines (Biological evaluation of medical devices; Part 5: Tests for in vitro cytotoxicity; Part 12: Biological evaluation of medical devices, sample preparation and reference materials (ISO 10,993–5:2009 and ISO/IEC 17,025:2005). A standard protocol for the NR assay was used from Nature Protocol^52^. MEMα supplemented with 10% FBS, 2 mM L-glutamine and suitable amount of antibiotics (amphotericin B, gentamycin) was used for the experiment. Also Zn(II) salt solutions were checked to eliminate potential cytotoxic effect of metal ions. Stock peptide solutions were prepared in H_2_O and then 100 times diluted in the medium. After adding proper mixtures of testing compounds and cells (1 × 10^5^ cells/mL) into each well, plates were incubated for 48 and 72 h in 5% CO_2_ at 37 °C . Next, medium was removed and 100 µL of NR solution (40 µg/mL) was added to each well followed by incubation for 2 h at 37 °C. After removing the dye, wells were rinsed with PBS and left to dry. Then, NR destain solution (1% glacial acetic acid, 50% of 96% ethanol and 49% of deionized water; v/v) was added to each well. The plates were shaken (30 min, 500 rpm) until NR was extracted from the cells and formed a homogenous solution. The absorbance was measured using microplate reader at 540 nm. As a negative control untreated cells were considered as 100% of potential cellular growth. Furthermore, cells incubated with 1 µM staurosporine were used as a positive control.

### AFM imaging

All compounds before preparation were diluted in HFIP and HFIP was left to evaporate overnight. Dry film was diluted in Mili-Q water to 0.5 mM concentration and pH was set to 7.4. The molar ratio of metal ions to peptides was 1:1. The samples were incubated at 298 K and AFM images were taken after 24 h and 7 days. AFM imaging was performed according to previously described procedure^53^ using a Veeco Dimension V atomic force microscope in a tapping mode, on mica plates. Images of most representative structures are shown. The images were processed with Nanoscope software and for height distribution analysis WSxM software was used.

## Supplementary Information

Supplementary Information.

## Data Availability

The datasets used in the current study available from the corresponding author upon request.
