# Supplementary material for: Zn(II) binding to pramlintide results in a structural kink, fibril formation and antifungal activity
Source: Sci Rep. 2022 Nov 29;12:20543. doi: 10.1038/s41598-022-24968-y (PMC9708664; doi:10.1038/s41598-022-24968-y)
Supplement: Supplementary file 1 — Supplementary Information. [file 41598_2022_24968_MOESM1_ESM.docx]

## Electronic Supplementary Information for:

Zn(II) binding to pramlintide results in a structural kink, fibril formation and antifungal activity

Dorota Dudek^1^, Emilia Dzień^1^, Joanna Wątły^1^, Aleksandra Mikołajczyk^2^, Agnieszka Matera-Witkiewicz^2^, Agata Hajda^3^, Joanna Olesiak-Bańska^3^ and Magdalena Rowińska-Żyrek^1*^

^1^ Faculty of Chemistry, University of Wrocław, F. Joliot-Curie 14, 50-383 Wrocław, Poland
^2^ Screening of Biological Activity Assays and Collection of Biological Material Laboratory, Wroclaw Medical University Biobank, Faculty of Pharmacy, Wrocław Medical University, Poland
^3^ Faculty of Chemistry, Wrocław University of Science and Technology, Wyb. Wyspiańskiego 27, 50-370 Wrocław, Poland


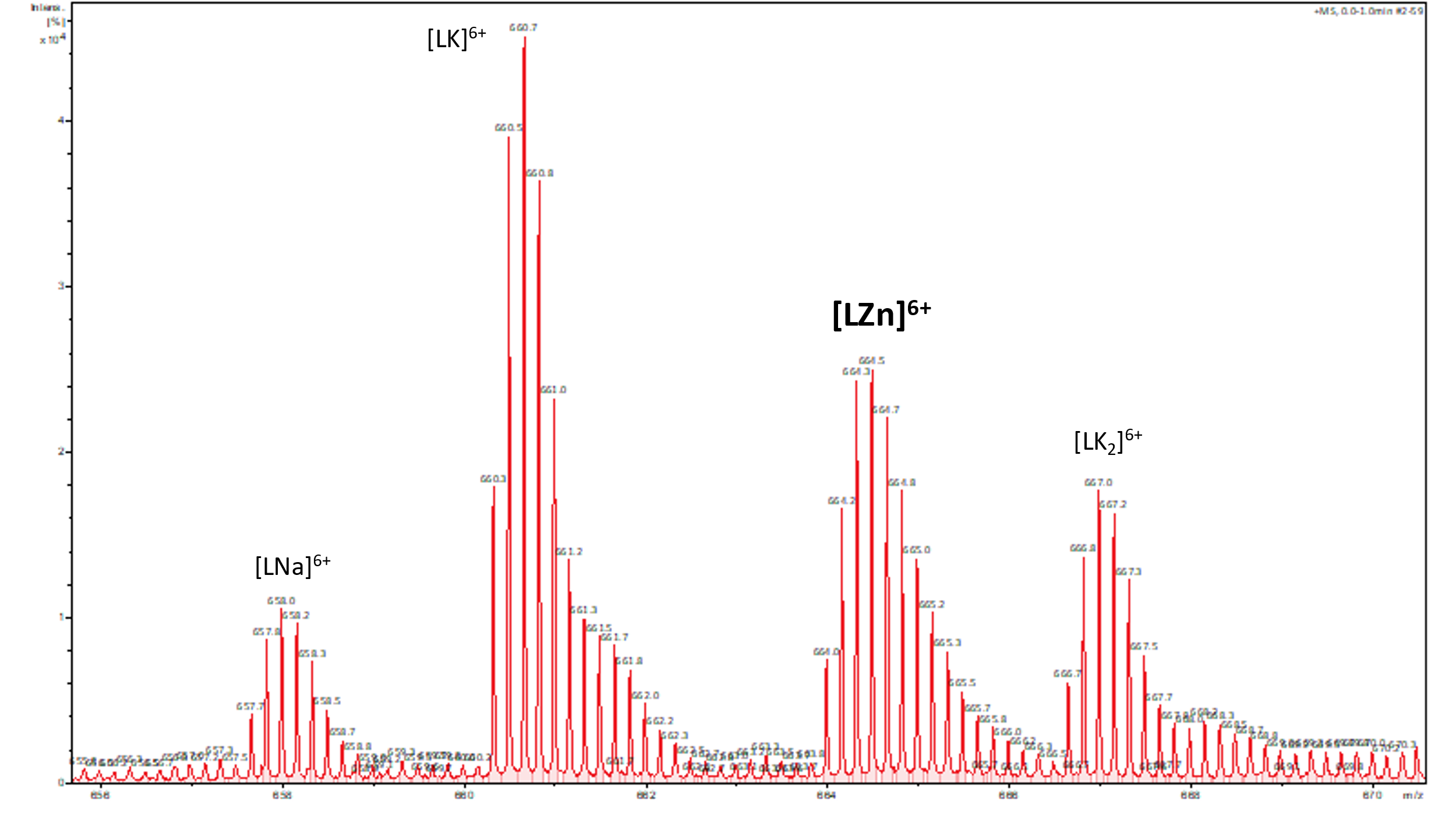


**Figure S1.** ESI-MS spectra of Zn(II)-rat amylin complex.

**A**
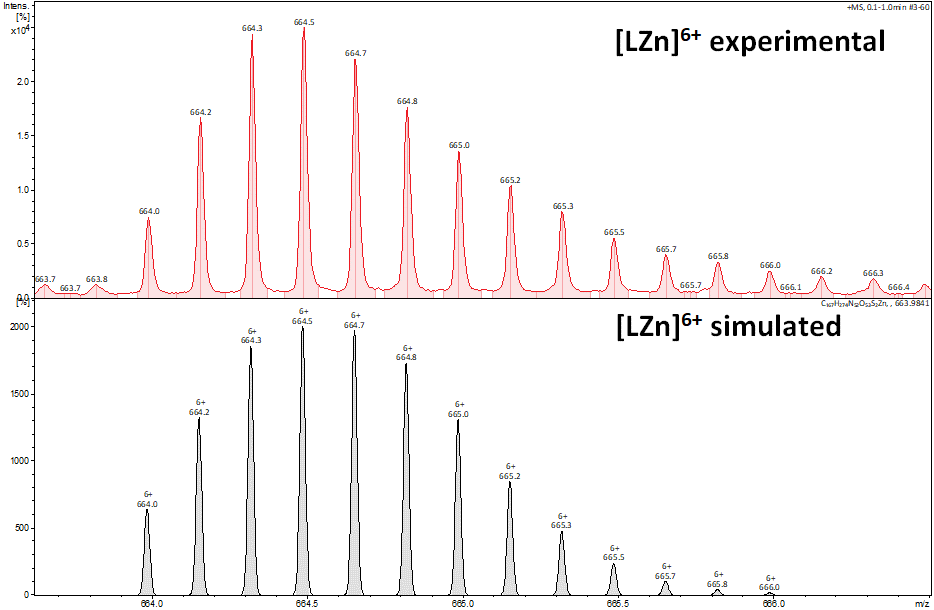


**B**


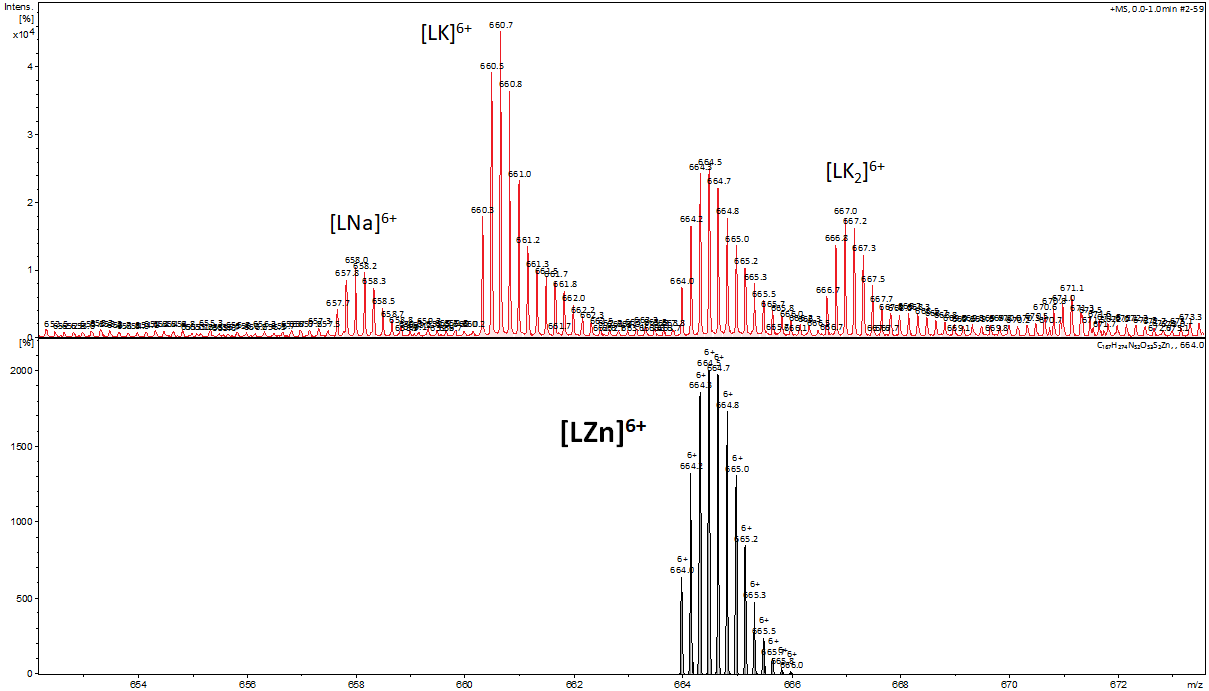


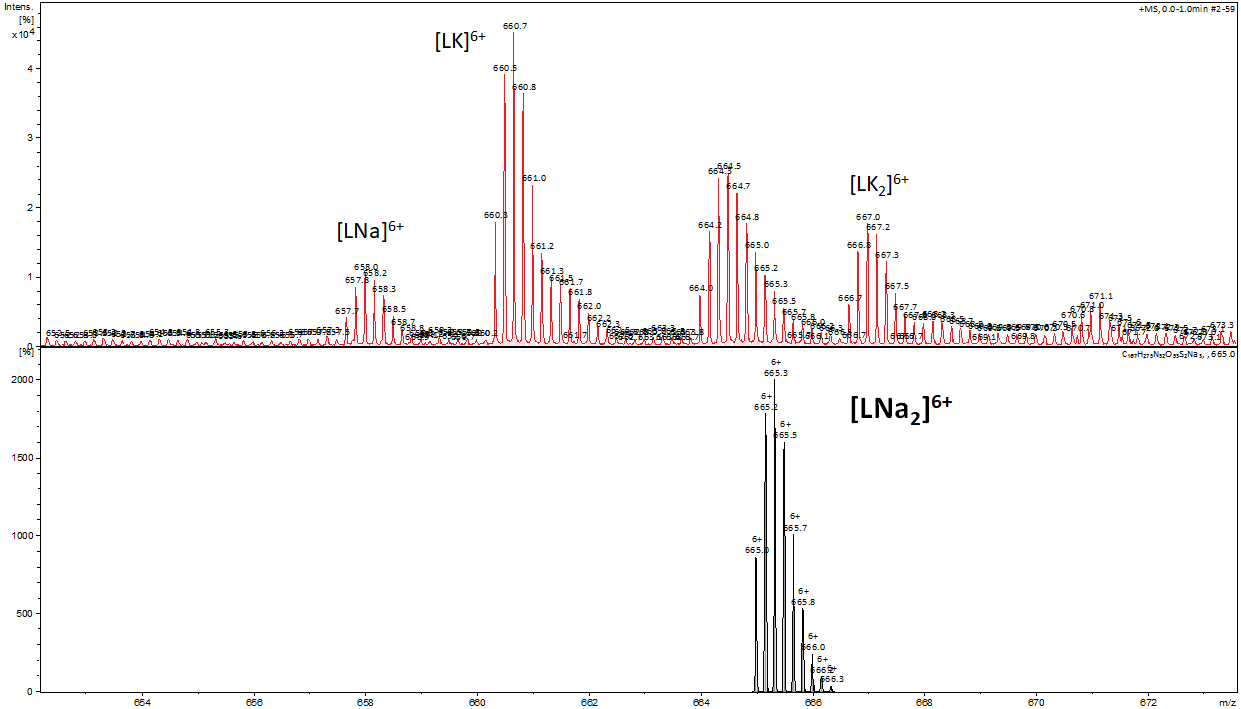


**Figure S2**. A) Isotopic distribution of Zn(II)-rat amylin complex; B) Simulated signals of the zinc-rat amylin complex are overlapped with the two sodium adduct of rat amylin

**Table S1**. Potentiometric data for proton and Zn(II) complex with rat amylin.

|  | **Rat amylin** | |
| --- | --- | --- |
|  | KCNTATCATQRLANFLVRSSNNLGPVLPPTNVGSNTY-NH_2_ | |
| **species** | **log**β | **pK_a_** |
| HL | 10.20 (4) | 10.20 (Lys) |
| H_2_L | 19.69 (3) | 9.49 (Tyr) |
| H_3_L | 26.82 (6) | 7.13 (N-t) |
| **Zn(II) complex** |  |  |
| ZnL | 7.56 | - |
| ZnH_-1_L | -1.20 | 8.76 |
| ZnH_-2_L | - | - |
| ZnH_-3_L | -23.27 | - |

**Figure S3.** Distribution diagrams for the formation of Zn(II) complex with rat amylin. Conditions: T = 298 K, [Zn(II)] = 0,45x10-3 M; Zn(II)/L molar ratio = 0.9:1.

***In vitro* toxicity studies**

The cytotoxic effect of compounds selected after the antimicrobial testing (which was 256 μg/mL of amylin_1-19_ and zinc complexes of amylin_1-19_ and pramlintide) was determined. The NR cytotoxicity assay was performed using RPTEC cell line from ECACC collection. This method allows to determine the cells viability using their ability to incorporate and bind neutral red dye in lysosomes.^1^ No significant decrease in cell viability was detected after 48 and 72 h incubation with the Zn(II)-pramlintide complex (viability equaled 96% and 99%, respectively).

**References**

1. Repetto, G., del Peso, A. & Zurita, J. L. Neutral red uptake assay for the estimation of cell viability/cytotoxity. *Nat Protoc.* **3**(7), 1125-1131 (2008).
